# Targeting inflammatory factors for chemoprevention and cancer interception to tackle malignant mesothelioma

**DOI:** 10.18632/oncoscience.605

**Published:** 2024-05-23

**Authors:** Joseph R. Testa, Yuwaraj Kadariya, Joseph S. Friedberg

**Affiliations:** ^1^Cancer Prevention and Control Program, Fox Chase Cancer Center, Philadelphia, PA 19111, USA; ^2^Department of Surgical Oncology, Fox Chase Cancer Center, Philadelphia, PA 19111, USA

**Keywords:** asbestos, *Bap1* mutations, chemoprevention agents, inflammation, preclinical genetically engineered mouse models

## Abstract

Mesothelioma is an incurable cancer of the mesothelial lining often caused by exposure to asbestos. Asbestos-induced inflammation is a significant contributing factor in the development of mesothelioma, and genetic factors also play a role in the susceptibility to this rapidly progressive and treatment-resistant malignancy. Consequently, novel approaches are urgently needed to treat mesothelioma and prevent or reduce the overall incidence of this fatal disease. In this research perspective, we review the current state of chemoprevention and cancer interception progress in asbestos-induced mesothelioma. We discuss the different preclinical mouse models used for these investigations and the inflammatory factors that may be potential targets for mesothelioma prevention. Preliminary studies with naturally occurring phytochemicals and synthetic agents are reviewed. Results of previous clinical chemoprevention trials in populations exposed to asbestos and considerations regarding future trials are also presented.

## INTRODUCTION

Mesothelioma is an incurable cancer of the mesothelial lining of the pleura, peritoneum, pericardium, and testes. Both environmental and genetic risk factors influence disease susceptibility [1]. Asbestos-induced inflammation and DNA damage play critical roles in mesothelioma pathogenesis, which transpires during a latency period of several decades. Patients with mesothelioma, particularly those with the pleural form of the disease (MPM), are often surgically inoperable and refractory to standard therapy. Immunotherapies have become a standard treatment for MPM patients. Still, the durability of most therapeutic responses remains short and inevitably results in relapse, with no second line of treatment available [2]. Consequently, novel, innovative approaches are urgently needed to improve therapies and reduce the overall incidence of mesothelioma. Early interventions would include the use of certain drugs or other substances to prevent cancer in individuals who are at high risk of developing the disease (chemoprevention) and actively intercepting a malignant process before the full-blown advanced tumor presents in the clinic (“cancer interception”) [3].

Mesothelioma carcinogenesis has been proposed to be associated with asbestos-induced mesothelial cell necrosis that leads to the release of high-mobility group protein B1 (HMGB1), a mediator of inflammation that activates the NLRP3 inflammasome and subsequent IL-1β secretion, as well as macrophage accumulation and the release of TNF-α, which increases the survival of asbestos-damaged mesothelial cells [4]. As a result of these aggregate events, critical genetic alterations are thought to accumulate within the affected mesothelial cells, initiating the onset of mesothelioma [5].

Three of the most frequent clonal genetic alterations that occur in human mesothelioma cells are somatic mutations and deletions of the tumor suppressor genes *BAP1*, *CDKN2A/B*, and *NF2* [6], and germline mutation of *BAP1* is a well-established genetic risk factor for mesothelioma [7]. In addition to IL-1β and TNF-α, other inflammatory cytokines, such as IL-6, are released from mesothelial cells and macrophages following asbestos exposure [8]. TNF-α activates the NF-κB pathway, resulting in mesothelial cell survival and resistance to the cytotoxic effects of asbestos. Consequently, NF-κB signaling may represent a potential molecular target for mesothelioma prevention, cancer interception, and therapy.

The NRF2/MAPK signal transduction pathway has been identified as another potentially critical target for prevention or interception in mesothelioma [9]. Activation of nuclear factor erythroid 2-related factor 2 (NRF2) signaling is regulated by asbestos-induced oxidative stress and the release of reactive oxygen species (ROS), which causes dissociation of NRF2 from inhibitory Keap1 and subsequent translocation of free NRF2 into the nucleus, where it binds to the antioxidant response element (ARE), resulting in transcription of antioxidant and detoxifying genes. In addition, cytosolic NRF2 is a downstream effector of activated ERK, JNK, and p38. Various anti-cancer phytochemicals have been reported to hold promise for the chemoprevention/interception of mesothelioma, based mainly on their activity in cell culture assays, and many of these natural substances act directly or via crosstalk with the NRF2/MAPK pathway [9]. These promising phytochemicals include anacardic acid, apigenin, curcumin, gallic acid, quercetin, sulforaphane, and ursolic acid. Furthermore, a standardized extract of the *Filipendula vulgaris* plant, known as dropwort, significantly reduced mesothelioma cell proliferation, viability, migration, and *in vivo* tumor growth [10]. Notably, inactivation of Hippo pathway components NF2 and LATS1/2 are common in mesothelioma and result in constitutive activation of the YAP1/TAZ transcriptional coactivators, thereby conferring malignant phenotypes to mesothelial cells. Since dropwort treatment was found to promote YAP and TAZ protein ubiquitination [10], this natural compound may represent a promising chemopreventive agent for targeting the Hippo pathway in individuals at risk of mesothelioma. The trace element selenium has also been implicated as a chemopreventive agent in various animal models. It was shown to inhibit cell growth and induce apoptosis in a dose-dependent manner in mesothelioma cells while having minimal effects on normal mesothelial cells [11]. Selenium has antioxidant properties that help to break down peroxides that can damage tissues and DNA and lead to inflammation. However, whether any of the above natural substances might be effective in mesothelioma prevention is unknown, as they have yet to be tested as preventive agents in laboratory animals or humans.

However, a population-based cancer prevention program was conducted for retinol (vitamin A) in workers previously exposed to crocidolite asbestos at Wittenoom, Western Australia [12]. The study began in 1990 and was published two decades later. Former asbestos workers at Wittenoom were separated into two groups: one was provided with retinol supplements (25,000 IU/day), and the second was given no supplements. The results of the investigation provided no support for possible preventive effects of retinol against mesothelioma in workers exposed to crocidolite.

Several synthetic agents, such as nonsteroidal anti-inflammatory drugs (NSAIDs), have been tested *in vivo* in mesothelioma animal models. In one investigation, the NSAID aspirin inhibited the carcinogenic effects of HMGB1, an inflammatory molecule implicated in mesothelioma tumor initiation and progression [13]. Aspirin and BoxA, a specific inhibitor of HMGB1, diminished mesothelioma growth in xenograft mice and significantly prolonged the survival of treated animals. On the other hand, Robinson et al. observed little benefit of aspirin in their asbestos-exposed MexTAg mouse model that expresses SV40 large T antigen (TAg) in the mesothelial compartment [14]. MexTAg mice given aspirin daily in the feed at 50 mg/kg or 250 mg/kg had a small but significant effect on disease latency (the time between asbestos exposure and the first evidence of disease). Still, they did not show a change in mesothelioma tumor incidence rate or an increase in the time that mice survived compared with control mice. Furthermore, in a parallel study of a human cohort of 1738 crocidolite-exposed people living or working in Wittenoom, Western Australia, individuals who reported use of aspirin or COX-2 inhibitors or both agents did not have a lower incidence of mesothelioma than a control group [14].

For chemoprevention/interception studies, our group has used preclinical models that recapitulate the genetic profile and phenotype of human mesothelioma. To simulate the environmental and genetic factors involved in the human disease counterpart, we have used asbestos-exposed, genetically engineered mouse (GEM) models that harbor germline heterozygous deletions or mutations of *Bap1*, *Nf2*, and/or *Cdkn2a*. For example, in one experiment, *Nf2*^+/−^;*Cdkn2a*^+/−^ mice were chronically exposed to asbestos in the presence or absence of the IL-1R antagonist anakinra. Although all mice in both experimental arms of this accelerated model of mesothelioma eventually developed tumors, anakinra-treated animals showed a significantly delayed median time of mesothelioma onset compared with placebo-treated mice (33 weeks vs. ~22.5 weeks, respectively; *P* < 0.0001). These and other findings from this study linked inflammation-related IL-1β/IL-1R signaling with the development of asbestos-induced mesothelioma. They provided the rationale for chemoprevention approaches targeting IL-1β/IL-1R signaling in populations at high risk of mesothelioma due to asbestos exposure. These populations include not only individuals exposed to asbestos occupationally or in the home but also people living in areas where rocks or soil contain erionite or other naturally occurring carcinogenic elongated mineral particles (EMP), such as in specific locations in Cappadocia, Turkey, North Dakota and Southern Nevada, USA [1], and Auckland, New Zealand [15].

To assess whether the inflammatory factor IL-6 might represent another potential prevention target in asbestos-induced mesothelioma, we tested the efficacy of SC144. This compound binds to glycoprotein 130 (gp130), the signal transducer of the IL-6/STAT3 signaling axis [16]. Treatment with SC144 inhibits the interaction between gp130 and IL-6 receptor (IL-6R), effectively blunting signaling from this inflammatory axis and inhibiting the expression of downstream STAT3 target genes [17]. Asbestos-exposed *Nf2*^+/−^;*Cdkn2a*^+/−^ mice chronically treated with SC144 showed significantly prolonged survival compared to asbestos-exposed vehicle-treated mice. STAT3 activity was markedly decreased in mesothelioma specimens from SC144-treated mice, and *in vitro* treatment of mesothelioma cells with SC144 markedly reduced the expression of the STAT3 downstream effectors, cyclin D1 and survivin [17].

While these synthetic agents effectively delayed tumor onset in asbestos-exposed *Nf2*^+/−^;*Cdkn2a*^+/−^ mice, mesothelioma was not prevented. One reason is that this mouse model is quite aggressive. In retrospect, a more appropriate mesothelioma mouse model for chemoprevention or interception studies would have a germline mutation in only a single tumor suppressor gene, e.g., asbestos-exposed *Nf2*^+/−^ or *Bap1*^+/−^ mice, which develop mesothelioma after a longer time frame than *Nf2*^+/−^;*Cdkn2a*^+/−^ animals.

Currently, there is no preventive intervention available for people who are at risk of developing mesothelioma, which is essentially any person with a history of exposure to asbestos or other EMPs or with a genetic risk factor (see below). Clinicians specializing in the treatment of mesothelioma often encounter members of a mesothelioma patient’s family who share the same asbestos exposure history but have not developed cancer to date. Similarly, high-risk workers, such as pipe fitters, often present for evaluation when a co-worker has developed mesothelioma. Tragically, other than radiographic surveillance and waiting to see if this terminal cancer develops, there is nothing to offer these individuals. Thus, developing efficacious strategies to prevent mesothelioma is a pressing unmet need.

Any agent used to prevent mesothelioma in humans must be administered for decades. Therefore, the ideal preventive agent not only needs to demonstrate efficacy in preventing mesothelioma but must also possess an extraordinary safety profile. The agent must also be affordable and administered orally for practical purposes.

We are currently using one such agent, sulforaphane, a natural compound found in cruciferous vegetables such as broccoli that is an indirect antioxidant with cytoprotective properties, to test its efficacy in preventing mesothelioma in asbestos-exposed *Bap1*^+/−^ mice. In addition to the environmental risk posed by asbestos and other carcinogenic mineral fibers, mesothelioma is associated with genetic risk factors, specifically inherited pathogenic mutations of *BAP1* [7] and other genes mainly involved in DNA damage repair [18–22]. Betti et al. reported that mesothelioma patients with pathogenic germline variants in *BAP1*, *CDKN2A*, or DNA repair genes had lower asbestos exposures than mesothelioma patients without germline variants in these genes or 94 other cancer-predisposing genes (*P* = 0.00002). They concluded that sensitivity to asbestos is increased in mesothelioma patients with pathogenic germline mutations in *BAP1* or other DNA repair genes [23]. Mice with germline *Bap1* heterozygous mutations have enhanced susceptibility to mesothelioma upon minimal exposure to crocidolite [24, 25] or chrysotile asbestos [25]. Chrysotile fibers have been shown to induce mesotheliomas characterized by an immunosuppressive tumor microenvironment comparable to that observed in most human mesotheliomas [25]. If our ongoing preclinical studies of sulforaphane in asbestos-exposed *Bap1*-mutant mice are successful, they will help inform the design of a preventive regimen for occupationally exposed asbestos workers at increased risk for mesothelioma and populations residing in areas heavily contaminated with asbestos, erionite, or other EMPs. Positive results could potentially also help in establishing future clinical guidelines for the long-term management of BAP1-tumor predisposition syndrome families, who are at high risk of mesothelioma and other cancers [26].

In 2012, Neri et al. reviewed the literature on chemoprevention trials in individuals exposed to asbestos, reporting a lack of relevant new chemoprevention trials since the mid-1990s and expressing an urgent need for research in this field [27]. Most trials of this nature ended after the beta-carotene and retinol efficacy trial (CARET) for lung cancer that had enrolled more than 18,300 smokers, former smokers, and asbestos-exposed workers who received a combination of 30 mg of beta-carotene and 25,000 IU of retinol per day or placebo [28]. After an average of 4 years of supplementation, the combination of beta-carotene and retinol had no benefit. Instead, it caused an increased incidence of deaths due to lung cancer and cardiovascular disease. Furthermore, after prolonged follow-up, there was an increased risk of mesothelioma in the active treatment arm versus the placebo arm among asbestos-exposed individuals (RR = 1.51; 95% CI = 0.80–2.84) [27]. Despite these setbacks, the existence of many agents and new signaling targets provide a reason for optimism in the future, keeping in mind Omenn’s lessons from the CARET trial, i.e., that “…design, conduct, documentation, relationships with participants, and preparedness for unexpected findings are all critical in chemoprevention research” [29].

## Abbreviations

ARE: antioxidant response element
*BAP1*: BRCA1 associated protein-1 gene
CARET: beta-carotene and retinol efficacy trial
*CDKN2A/B*: cyclin-dependent kinase inhibitor 2A/B genes
EMP: elongated mineral particles
ERK: extracellular signal-regulated kinases
GEM: genetically engineered mouse
gp130: glycoprotein 130
HMGB1: high-mobility group protein B1
IL-1β: interleukin-1 beta
IL-1R: interleukin-1 receptor
IL-6: interleukin-6
JNK: c-Jun N-terminal kinases
MAPK: mitogen-activated protein kinase
MPM: malignant pleural mesothelioma
*NF2*: neurofibromatosis 2 gene
NF-κB: nuclear factor kappa-light-chain-enhancer of activated B cells
NLRP3: NLR family pyrin domain containing 3 protein
NRF2: nuclear factor erythroid 2-related factor 2
NSAIDs: nonsteroidal anti-inflammatory drugs
p38: p38 mitogen-activated protein kinase
ROS: reactive oxygen species
RR: relative risk (risk ratio)
STAT3: signal transducer and activator of transcription 3
TAg: large T antigen
TAZ: transcriptional coactivator with PDZ-binding motif
TNF-α: tumor necrosis factor-alpha
YAP: yes-associated protein 1
